# Physician- and Patient-Elicited Barriers and Facilitators to Implementation of a Machine Learning–Based Screening Tool for Peripheral Arterial Disease: Preimplementation Study With Physician and Patient Stakeholders

**DOI:** 10.2196/44732

**Published:** 2023-11-06

**Authors:** Vy Ho, Cati Brown Johnson, Ilies Ghanzouri, Saeed Amal, Steven Asch, Elsie Ross

**Affiliations:** 1 Division of Vascular Surgery Department of Surgery Stanford University School of Medicine Stanford, CA United States; 2 Division of Primary Care and Population Health Department of Medicine Stanford University School of Medicine Stanford, CA United States; 3 College of Engineering Northeastern University Boston, MA United States; 4 Center for Innovation to Implementation Veterans Affairs Palo Alto Healthcare System Palo Alto, CA United States

**Keywords:** artificial intelligence, cardiovascular disease, machine learning, peripheral arterial disease, preimplementation study

## Abstract

**Background:**

Peripheral arterial disease (PAD) is underdiagnosed, partially due to a high prevalence of atypical symptoms and a lack of physician and patient awareness. Implementing clinical decision support tools powered by machine learning algorithms may help physicians identify high-risk patients for diagnostic workup.

**Objective:**

This study aims to evaluate barriers and facilitators to the implementation of a novel machine learning–based screening tool for PAD among physician and patient stakeholders using the Consolidated Framework for Implementation Research (CFIR).

**Methods:**

We performed semistructured interviews with physicians and patients from the Stanford University Department of Primary Care and Population Health, Division of Cardiology, and Division of Vascular Medicine. Participants answered questions regarding their perceptions toward machine learning and clinical decision support for PAD detection. Rapid thematic analysis was performed using templates incorporating codes from CFIR constructs.

**Results:**

A total of 12 physicians (6 primary care physicians and 6 cardiovascular specialists) and 14 patients were interviewed. Barriers to implementation arose from 6 CFIR constructs: complexity, evidence strength and quality, relative priority, external policies and incentives, knowledge and beliefs about intervention, and individual identification with the organization. Facilitators arose from 5 CFIR constructs: intervention source, relative advantage, learning climate, patient needs and resources, and knowledge and beliefs about intervention. Physicians felt that a machine learning–powered diagnostic tool for PAD would improve patient care but cited limited time and authority in asking patients to undergo additional screening procedures. Patients were interested in having their physicians use this tool but raised concerns about such technologies replacing human decision-making.

**Conclusions:**

Patient- and physician-reported barriers toward the implementation of a machine learning–powered PAD diagnostic tool followed four interdependent themes: (1) low familiarity or urgency in detecting PAD; (2) concerns regarding the reliability of machine learning; (3) differential perceptions of responsibility for PAD care among primary care versus specialty physicians; and (4) patient preference for physicians to remain primary interpreters of health care data. Facilitators followed two interdependent themes: (1) enthusiasm for clinical use of the predictive model and (2) willingness to incorporate machine learning into clinical care. Implementation of machine learning–powered diagnostic tools for PAD should leverage provider support while simultaneously educating stakeholders on the importance of early PAD diagnosis. High predictive validity is necessary for machine learning models but not sufficient for implementation.

## Introduction

Peripheral arterial disease (PAD) afflicts over 8 million Americans and is associated with an increased risk of major cardiac events, major limb events, and all-cause mortality [1]. In the current diagnostic approach, physicians perform an ankle brachial index (ABI) on patients in whom PAD is suspected based on risk factors or symptomatology; an ABI less than 0.9 is suggestive of PAD. Cross-sectional studies suggest PAD is underdiagnosed, with only 10%-30% of patients presenting with stereotypical symptoms and less than 50% of patients and primary care physicians reporting awareness of the disease [2,3].

Machine learning (ML) algorithms may improve PAD detection by identifying high-risk patients who would benefit from ABI testing. By integrating diverse data sources in the electronic health record, such as genomics, wearable data, and medical history, in nonlinear ways, ML may ease the cognitive workload of diagnosis while assisting clinical decision-making. Previously reported algorithms have demonstrated greater than 90% sensitivity and specificity, exceeding that of logistic regression [4,5].

Despite superlative diagnostic performance, previously reported barriers to ML implementation in health care include low acceptability among physicians due to alert fatigue and a lack of algorithmic transparency [6,7]. Patients have also voiced concerns that ML will interfere with the patient-physician relationship and increase the risk of data misuse or privacy violations [8,9]. Ultimately, improving PAD detection requires stakeholder acceptance of and investment in novel diagnostic approaches. A qualitative assessment of patients’ and physicians’ perceptions of a novel ML-powered diagnostic intervention for PAD is needed to better inform implementation strategies. In this study, we evaluate physician- and patient-elicited barriers and facilitators to the implementation of an ML-based PAD screening tool in outpatient clinics affiliated with a quaternary care teaching hospital.

## Methods

### Setting

This project was conducted jointly with Stanford University’s Divisions of Primary Care and Population Health, Vascular Medicine, and Cardiology. Interviews were conducted from September 2021 to May 2022. This quality improvement project received a nonresearch determination by the Stanford University Institutional Review Board (Eprotocol-62076).

We have previously described the development of an ML model based on the Stanford Medicine Research Data Repository, which contains clinical practice data from over 4 million adult patients from 1998 to 2020. This model outperformed Duval et al’s [10] traditional nomogram for PAD diagnosis and logistic regression with respect to sensitivity, specificity, and discrimination. The objective of this study is to solicit patients’ and physicians’ perspectives regarding the integration of this model into the electronic health record to notify physicians to consider PAD screening in patients with a high risk of PAD.

### Theoretical Framework

The Consolidated Framework for Implementation Research (CFIR) integrates metrics from previous implementation frameworks into 5 domains: intervention, outer setting, individual characteristics, inner setting, and process [11]. CFIR was chosen as the framework for this study because it allows identification of barriers and facilitators among diverse stakeholders and has been shown to be useful in guiding rapid-cycle evaluations of clinical interventions [12].

### Participants and Study Design

A semistructured interview guide was developed to contextualize the vignettes within barriers and facilitators from the CFIR domains. Vignettes and interview guides were pretested with 3 cardiovascular physicians who were excluded from the list of prospective interviewees to ensure appropriate clinical relevance and formatting. Vignettes were designed to simulate environments in which patients have a moderate pretest probability of PAD, with comorbidities that are established risk factors such as diabetes, hypertension, and old age. The order in which vignettes were administered was randomized between participants. An interview guide for physician participants containing patient vignettes and prompts is provided in Multimedia Appendix 1.

For the physician evaluation, semistructured interviews were conducted with faculty in cardiology, vascular medicine, and primary care to represent the variation of physicians who typically diagnose PAD. The study team sent an email to each department seeking volunteers for participation and arranged interviews with respondents. One author (VH) conducted interviews through videoconferencing with previous verbal consent.

**Figure 1:**
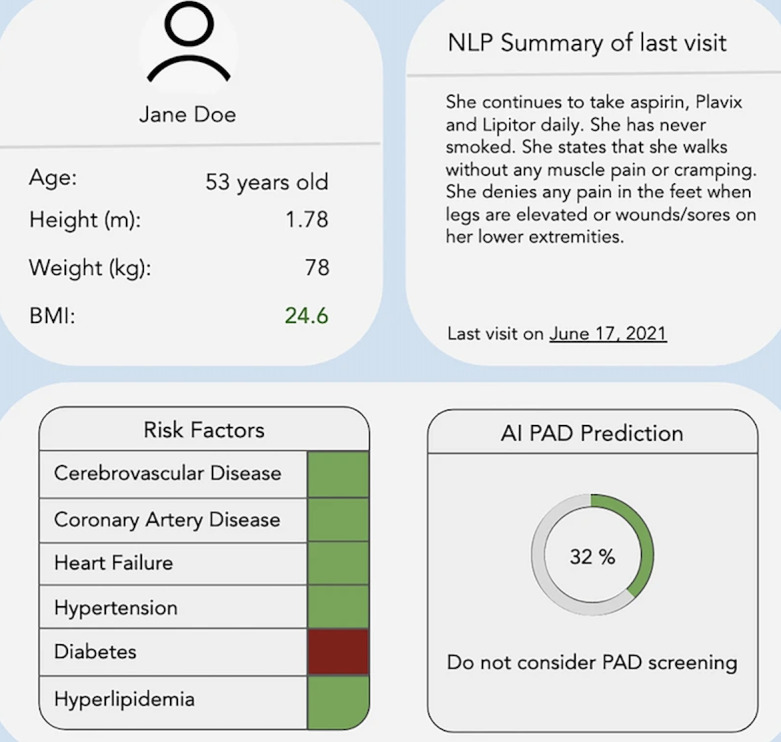
Peripheral arterial disease screening tool output presented to physician interviewees. NLP: natural language processing.

After discussing their current approach to diagnosing PAD, participants listened to a simulated patient vignette and were prompted to navigate an ML-powered dashboard containing the patient’s information and PAD risk prediction score while thinking aloud. A total of 2 simulated patient vignettes were used, one in which screening was recommended and one in which screening was not recommended. Figure 1 depicts the output of the PAD screening tool alongside summarized fictional patient data.

For the patient evaluation, participating cardiovascular physicians were asked for permission to contact patients who had been seen by them on an outpatient basis in the past 2 months. Physicians who gave verbal consent to proceed were also given the opportunity to identify patients who should not be contacted for study participation. A list of all eligible patients was then generated and randomized. Semistructured interviews were then sequentially conducted through telephone, with a total of 42 calls made without leaving voice messages to yield 14 patient interviews. One female researcher (VH) with previous postdoctoral clinical training in vascular surgery and no previous contact with study participants conducted interviews, prompting patients to discuss their current perceptions and previous experiences regarding ML and PAD with previous verbal consent. The researcher’s clinical background in vascular surgery was disclosed to physician interviewees but not to patient interviewees.

Interviews continued until thematic saturation was reached, defined as the inflection point after which new interviews ceased to surface new themes or perspectives. All interviews were performed with only the researcher and interviewee present, and no repeat interviews were performed. Transcripts were not made available to participants after the fact. An interview guide for patient participants is provided in Multimedia Appendix 2.

### Qualitative Analysis

As per standard rapid analytic methods, template summaries were used to summarize each interview transcript into structured one-page documents that captured major a priori themes [13,14]. Template summaries are frequently used in rapid qualitative analyses, allowing for an expedited review process without formal coding (Multimedia Appendix 3). Summaries were then analyzed with deductive and inductive approaches, allowing for subsequent organization by CFIR domain. Deductive themes were derived from outcomes of interest, while emergent barriers and facilitators were identified inductively. Subsequent analysis and reporting conformed to the COREQ (Consolidated Criteria for Reporting Qualitative Research) standardized guidelines (Multimedia Appendix 4).

### Ethical Considerations

This study was deemed not to constitute human participant research by the Stanford University institutional review board as a quality improvement study (IRB code 62076). All study data were anonymized and stored locally on an encrypted institutional device. All physician research participants were awarded a US $25 gift card for participation, while patient research participants were not offered any compensation.

## Results

A total of 12 physicians (6 primary care and 6 cardiovascular specialists) and 14 patients were interviewed. Table 1 provides key sample characteristics for participating physicians. There was an equal distribution of male and female physicians, with the majority of interviewees having less than 10 years of practice experience. Table 2 provides key sample characteristics for participating patients. The majority of patients were male, greater than 50 years of age, and used Medicare as their primary insurance plan.

Out of the 37 CFIR constructs, 5 emerged as barriers to implementation, 4 emerged as facilitators, and 1 construct had both barrier and facilitator attributes. Table 3 summarizes the relevant CFIR domains, constructs, and subthemes.

**Table 1:** Key sample characteristics for participating physicians.

Characteristics			Values, n (%)
**Gender**			
	Male	6 (50)	
	Female	6 (50)	
	Other or decline to state	0 (0)	
**Race**			
	Asian American	4 (33)	
	Hispanic or Latino	1 (8)	
	Non-Hispanic African American or Black	1 (8)	
	Non-Hispanic White	6 (50)	
**Highest level of postdoctoral education**			
	Residency	6 (50)	
	Fellowship	6 (50)	
**Medical practice (years)**			
	0-5	4 (33)	
	5-10	4 (33)	
	10-20	1 (8)	
	>20	3 (25)	

**Table 2:** Key sample characteristics for participating patients.

Characteristics		Values, n (%)
**Gender**		
	Male	9 (64)
	Female	5 (35)
	Other or decline to state	0 (0)
**Age (years)**		
	Less than 30	0 (0)
	30-50	2 (14)
	50-70	10 (71)
	>70	2 (14)
**Race**		
	Asian American	2 (14)
	Hispanic or Latino	3 (21)
	Non-Hispanic African American or Black	3 (21)
	Non-Hispanic White	6 (42)
**Primary insurance**		
	Private	4 (28)
	Medicare	10 (71)
	Other	0 (0)

**Table 3:** Patient and physician interview themes.

CFIR^a^ domain	Barriers	Facilitators
Intervention characteristics	Complexity: physicians’ and patients’ perceptions of machine learning as difficult Evidence strength and quality: lack of physician and patient awareness regarding PAD^b^	Intervention source: endorsement from vascular surgeons Patient preference for physicians to remain the primary interpreters of health care data Relative advantage: patients’ and physicians’ perceptions of machine learning as a useful decision-making adjunct
Inner setting	Relative priority: physician-reported low urgency regarding PAD screening	Learning climate: physician willingness to incorporate clinical decision support into workflows
Outer setting	—^c^	External policies and incentives: institutional support for precision medicine
Individual characteristics	Knowledge and beliefs about intervention: patient concerns regarding data security and privacy Individual identification with organization: specialty physicians’ perception that PAD management is not their responsibility	Knowledge and beliefs about intervention: physicians’ perceptions that an ML^d^-powered PAD tool would improve their ability to care for PAD patients

^a^CFIR: Consolidated Framework for Implementation Research.

^b^PAD: peripheral arterial disease.

^c^Not available.

^d^ML: machine learning.

### Intervention Characteristics Domain

Intervention source refers to the perception of key stakeholders regarding whether the intervention is externally or internally developed. Among physicians, primary care physicians responded positively to the affiliation of the study group within the Stanford University Division of Vascular Surgery. These participants felt that having specialists who frequently treat PAD involved in the implementation process demonstrated stakeholder investment that increased the legitimacy of the intervention.

If [the intervention] came from our vascular surgery team or someone that I trusted used it I’d think about implementing it.

Physician 3

Relative advantage is defined by stakeholders’ perceptions regarding the benefit of implementing the intervention against an alternative. Most physicians felt that the intervention would improve their ability to diagnose PAD.

While most patients were comfortable with their physician using this tool in their care, only many did not feel comfortable making decisions about their health based on an ML-powered tool alone. Many patients who were interested in making decisions based on the proposed intervention stipulated that they would want to ensure that their doctors agreed with the model’s recommendation, making their physician the primary interpreter of health care data and the ultimate decision maker regarding the conclusions of any proposed screening model.

I think artificial and human intelligence should be balanced, with 75% human and 25% artificial intelligence.

Patient 4

I think [the intervention] could start good conversations, and if there was something that it flagged I’d discuss it further with my physician.

Patient 12

Complexity refers to the perceived difficulty of the intervention. While few stakeholders had first-hand experience with ML, both providers and patients expressed concerns that the difficulty of performing ML tasks accurately could lead to unreliable results.

What goes into [the intervention]? I don’t like to take numbers and data without underlying evidence that this algorithm is validated.

Physician 3

I’ve heard about [machine learning], but for it to be used in healthcare it must be really mature… unless it’s very well trained and matured you cannot guarantee the results.

Patient 13

Aside from the technical complexity, patients also expressed concerns that the intervention could complicate the physician-patient relationship, creating opportunities for misunderstandings or mistakes in care coordination.

I could see [the intervention] being good in healthcare because it has the most up to date technology, but it could be bad… in that it changes your interaction with the doctor, or if the doctor doesn’t understand what [the intervention] is saying and the two aren’t communicating… that’s bad. There could be a glitch or misinterpretation.

Patient 1

Evidence strength and quality is a subdomain describing stakeholders’ perceptions of the validity of evidence supporting the intervention’s success. Most providers were not aware of guidelines advocating or discouraging testing patients without lower extremity symptoms for PAD.

I don’t think there’s really established guidelines for screening for asymptomatic PAD.

Physician 1

Only 1 provider directly referenced current guidelines and ultimately felt there was a potential benefit to PAD screening.

I think there is a potential benefit [to testing asymptomatic patients for PAD]. American College of Cardiology, American Heart Association and vascular surgery guidelines would say potential benefit… I think the United States Preventative Task Force would say it’s not clear if there’s a benefit.

Physician 11

Analogously, only 2 of the 14 interviewed patients were familiar with PAD. One patient was a retired physician, and the other had heard of PAD from friends who were in the health care industry.

### Inner Setting Domain

Relative priority entails stakeholders’ perceptions about the importance of the implementation. Among providers, most felt that early diagnosis of PAD was not urgent compared to other diseases for which screening is routinely performed. Of the 6 primary care doctors, 3 said that PAD was less urgent than cardiac disease.

[PAD] is unlike heart disease in that there’s such a thing as a heart attack, so missing screening for heart disease has grave implications. Patients who have risk factors for PAD typically have cardiovascular risk factors and are being treated aggressively anyway.

Physician 4

Similarly, 1 physician felt that they already had many tests to request of patients, such that PAD screening may not always feel appropriate:

I have to put [the] risk benefit ratio [of PAD screening] in the context of everything else. So if they haven’t had their colonoscopy, or their mammogram… do I send them for that if they have limited bandwidth?

Physician 4

Learning climate describes a setting in which stakeholders feel that there is enough time, space, and psychological safety to try new practices. Multiple physicians cited familiarity with similar clinical decision support interventions and a willingness to incorporate the intervention.

I know there’s tools like this and others being created for heart failure risk prediction, so I think it’s interesting how we can have these show up on schedules and outpatient records to help us more consistently screen people.

Physician 7

### Outer Setting Domain

External policies and incentives are strategies to spread interventions, including policies and regulations, external mandates, recommendations, and guidelines. Multiple physicians referenced broader initiatives at Stanford in precision medicine and artificial intelligence as a reason why they were familiar with and interested in the intervention.

Stanford has really gone in on precision medicine, you know finding ways to use technologies to assist us in doing our jobs. I haven’t been approached about such tools specifically before you but I think it’s good that there is a general enthusiasm about it and investment to bring this to reality.

Physician 1

### Individual Characteristics Domain

Knowledge and beliefs about the intervention reflect individual familiarity with facts, truths, and principles related to the intervention. All providers stated that they diagnosed PAD based on clinical suspicion driven by traditional risk factors such as hypertension, diabetes, smoking history, and symptoms including lower extremity pain or wounds. Most providers believed that PAD was relatively underdiagnosed; even providers who did not think the intervention would benefit their practice believed that patients were being missed based on current diagnostic approaches.

PAD, we didn’t get that much teaching on it. Everyone thinks so much about coronary artery disease and I feel PAD seems more subtle and we know less about it. I could tell you so much about [coronary artery disease] and I think I know less for PAD.

Physician 12

Furthermore, most providers had positive perceptions of ML in health care.

I’m all for machine learning in the record to help me be a better doctor. It’s going to help me not miss diseases, and its going to help me manage diseases better.

Physician 4

Patients’ perceptions of ML in health care were generally positive. Some patients associated ML and artificial intelligence with previous innovations they viewed favorably, including robotic surgery and learning software for autistic children.

I’m all for technology; I think I’ve heard about using artificial Intelligence to do surgery, and I don’t know much about it but I think it’s a good tool.

Patient 7

I have [artificial intelligence], I hire programmers, my kids use AI-powered software for their autism. I like AI.

Patient 6

Some patients objected to the phrase “artificial intelligence” and voiced concerns about its use by nonphysician entities.

The wording is scary. ‘Artificial intelligence’ sounds like it comes from aliens, like not human. The wording should be switched… how it comes off is very strange.

Patient 3

There’s a lot of potential really good stuff you can use machine learning for. On the other hand, if you put it in the hands of insurance companies for them to put together their predictive algorithms I think you may have issues.

Patient 15

Individual identification with an organization refers to how individuals perceive the organization and their relationship and degree of commitment with that organization. Among cardiovascular specialists, some providers felt that diagnosing PAD was the responsibility of primary care providers. This led to concerns regarding whether they would be open to using the intervention.

To take on PAD screening would be kind of an additional thing outside my normal workflow… I would prefer for the local physician to do the evaluation.

Physician 8

Conversely, primary care physicians cited a tension between specialists seeking to screen for a specific disease of interest and primary care physicians who are responsible for managing the whole patient:

No offense, but everybody comes to primary care and says, ‘Could you screen for my disease?’ Whether it be incontinence or prostate cancer, and then they want us to use a specific separate tool.

Physician 4

## Discussion

### Summary of Findings

In this qualitative analysis of patients’ and physicians’ attitudes toward the development of an ML-powered PAD diagnostic tool, barriers to implementation followed four interdependent themes: (1) low familiarity or urgency in detecting PAD; (2) concerns regarding the reliability of ML; (3) differential perceptions of responsibility for PAD care among primary care versus specialty physicians; and (4) patient preference for physicians to remain primary interpreters of health care data. Facilitators followed two interdependent themes: (1) enthusiasm for clinical use of the predictive model and (2) willingness to incorporate ML into clinical care.

Low physician and patient awareness of PAD is well documented. In separate surveys, 26% of patients expressed familiarity with PAD, while only 49% of physicians knew when their patients had a previous PAD diagnosis [3,15]. Physicians’ perceptions that PAD is not as serious as other cardiovascular diseases may fuel downstream care disparities; in a registry evaluation of over 68,000 outpatients with cardiovascular disease, patients with PAD were less likely to be receiving adequate risk factor management compared to patients with coronary or cerebrovascular disease [16]. Our findings suggest that these attitudes persist in a quaternary academic care setting, but there are also opportunities for stakeholder education given the interest expressed by multiple respondents in learning more about PAD. In our sample, physician awareness of PAD may be impacted by the extent of clinical experience, with most physicians having less than 10 years of clinical practice.

While stakeholders were generally interested in leveraging ML to identify patients with PAD, they sought assurances about the algorithm’s reliability and scope. Physicians requested accompanying citations and explanatory text about the algorithm’s development and accuracy; this feedback has since been incorporated into further iterations of the ML tool interface [5]. Patients stipulated that the tool should be an adjunct rather than a replacement for human judgment; one specifically disliked the term “artificial intelligence” because it implied that machines would outlearn and replace people. Emphasizing that doctors would be using the intervention as one of many diagnostic tools was central to patient acceptability, which has been similarly reported in qualitative studies soliciting patients’ perceptions of ML tools in general [17].

Physician interviews also revealed ambiguity regarding who should be responsible for diagnosing PAD. Primary care physicians reported less familiarity with PAD and difficulty balancing the need to screen and treat a wide variety of diseases. Cardiovascular specialists were more knowledgeable about PAD but felt that the diagnosis was better left to the primary care physicians. While ambiguity regarding the practice domain of generalists and specialty providers is often influenced by cultural norms, patient comorbidities, and local resources, facilitating communication between specialists who suspect PAD and their primary care providers may improve diagnosis rates [18,19].

Facilitators for implementation included institutional and interventional support for improved methods of PAD diagnosis. In 2015, Stanford Medicine introduced a precision health framework reflecting a strategic focus toward leveraging data science, ML, and predictive analytics into clinical care. Institutional investment in these methods, in addition to endorsement of the algorithm from our Division of Vascular Surgery, which specializes in medical and surgical management of patients with PAD, were perceived as facilitators by stakeholders.

This study had several limitations. First, our sample was limited to a single quaternary academic center, which may limit the broad applicability of the results. However, interviewees included physicians and patients across departments, providing a rich perspective from multiple specialties. Second, since interviews were performed on a voluntary basis, it is possible that stakeholders who did not volunteer would have different perceptions of the intervention. However, interviews were conducted until thematic saturation, providing as broad a range of viewpoints as possible. Third, limited participant demographic information was collected as part of this quality improvement project. While identifying a patient’s primary insurance provider offers some insight into their socioeconomic status, there are many other variables that influence patients’ perceptions of PAD, ML, and the subsequent acceptability of the proposed intervention.

### Conclusion

In this qualitative analysis of patients’ and physicians’ attitudes toward the development of an ML-powered PAD diagnostic tool, barriers to implementation followed four interdependent themes: (1) low familiarity or urgency in detecting PAD; (2) concerns regarding the reliability of ML; (3) differential perceptions of responsibility for PAD care among primary care versus specialty physicians; and (4) patient preference for physicians to remain primary interpreters of health care data. Facilitators followed two interdependent themes: (1) enthusiasm for clinical use of the predictive model and (2) willingness to incorporate ML into clinical care. Implementation of ML-powered diagnostic tools for PAD should leverage institutional and interventional support while simultaneously educating stakeholders on the importance of early PAD diagnosis.

## Appendix

Multimedia Appendix 1 Interview guide for physicians participating in the study.

Multimedia Appendix 2 Interview guide for patients participating in the study.

Multimedia Appendix 3 Template summary example of de-identified participant quotations contextualized in study themes and subthemes.

Multimedia Appendix 4 Checklist designating manuscript conformation to Consolidated Criteria for Reporting Qualitative Research standardized guidelines (COREQ).

## Abbreviations

ABI: ankle brachial index
CFIR: Consolidated Framework for Implementation Research
COREQ: Consolidated Criteria for Reporting Qualitative Research
ML: machine learning
PAD: peripheral arterial disease

## Data Availability

The data sets generated during or analyzed during this study are available from the corresponding author on reasonable request.
