# Supplementary material for: Physician- and Patient-Elicited Barriers and Facilitators to Implementation of a Machine Learning–Based Screening Tool for Peripheral Arterial Disease: Preimplementation Study With Physician and Patient Stakeholders
Source: JMIR Cardio. 2023 Nov 6;7:e44732. doi: 10.2196/44732 (PMC10660241; doi:10.2196/44732)
Supplement: Multimedia Appendix 1 [file cardio_v7i1e44732_app1.docx]

**PAD ML Interview Guide: Staff & Physicians**

**RESEARCH QUESTIONS** *(for internal purposes only)*

What are staff’s perceptions regarding barriers and facilitators to machine-learning based PAD screening implementation, particularly in regards to usability, acceptability, and compatibility with patient needs?

**STAFF DEMOGRAPHIC DATA**

| **Staff ID** |  |
| --- | --- |
| **Subspecialty** |  |
| **Title** |  |

***3 people in a group minimum before identification, otherwise collapse.**

***Let people know who is going to know they participate or not**

***Check color for color blind individuals (gradations grey or black and grey)**

**INTRODUCTION**

Hi, my name is XXXXXX and I am calling from Stanford University. I am working with Stanford Vascular Surgery on a project to evaluate the implementation of a PAD screening tool. Today, we want to learn about your ideas regarding a PAD screening tool.

You are also free to skip questions at any time. These conversations usually take around **15 to 20 minutes**. Is this a good time for you to chat?

Before we start, everything you say today **will be kept confidential. I am the only person who will be looking directly at transcripts, and will not tell anyone whether you participated or not**.

I would also like to audio record the interview to help me focus on you instead of taking notes. Are you comfortable with me **recording** our confidential discussion?

- Ok, I am **turning on the recorder** now.
- Do you give your **consent** to be recorded to this evaluation?
- Can you **please say your name, date**, and that you **accept being recorded**?
- To be **cognizant of your time**, how long are you able to chat with me today? Do you have a hard stop we need to pay attention to?

**STAFF PRIOR EXPERIENCES WITH PATIENTS WITH PERIPHERAL ARTERIAL DISEASE**

1. **Tell me about how you diagnose peripheral arterial disease. What signs, symptoms, and factors do you keep in mind?**
2. **Approximately how many times a month do you decide to screen a patient for peripheral arterial disease?**
3. **Can you please tell me about any predictive or machine learning models you currently use clinically, if any?**

**TOOL WALKTHROUGH #1 (POS FIRST), FOR ODD NUMBERED STUDY IDS**

**I am going to show you two versions of a tool designed to advise physicians on when to screen for peripheral arterial disease. First, I’ll read the following vignette:**

**John D. is a 53 year old obese male who presents to your clinic for a well visit. He has a history of stroke, myocardial infarction, and three-drug hypertension.**

**I’ve given you control of the mouse, and you can now click ‘First Dashboard’ on the Home Screen. Click the links as you see fit, and please voice your reactions and thought process aloud. We particularly appreciate critical feedback to improve our interface.**

**I’d like to hear your thoughts about this specific dashboard.**

1. **What did you like about this dashboard?** *Usability*
2. **What could be improved about this dashboard?** *Usability*
3. **How did you feel about the information provided on the dashboard? Were there any items that felt irrelevant, or items you wish you saw?**

**Thank you for your feedback! Please hit the left button until you return to the first screen you saw. I will now read this vignette:**

**Jana D. is a 53 year old female who presents to your clinic for a well visit. She has a history of diabetes well controlled on Metformin.**

**Please click ‘Second Dashboard’ on the Home Screen. Again, click the links as you see fit, and please voice your reactions and thought process aloud. We particularly appreciate critical feedback to improve our interface.**

1. **What did you like about this dashboard?** *Usability*
2. **What could be improved about this dashboard?** *Usability*
3. **How did you feel about the information provided on the dashboard? Were there any items that felt irrelevant, or items you wish you saw?** *Acceptability*

**I will now ask you about your impressions of the entire tool as a whole, independent of how the information provided was arranged.**

1. **How do you think this PAD screening tool would influence your recommendations to patients?** *Patient needs & resources*
2. **What benefits would you anticipate to using this screening tool? What challenges?** *Acceptability*

**TOOL WALKTHROUGH #2 (NEG FIRST) FOR EVEN NUMBER STUDY ID’S**

**I am going to show you two versions of a tool designed to advise physicians on when to screen for peripheral arterial disease. First, I’ll read the following vignette:**

**John D. is a 53 year old obese male who presents to your clinic for a well visit. He has a history of diabetes, well controlled on Metformin.**

**I’ve given you control of the mouse, and you can now click ‘First Dashboard’ on the Home Screen. Click the links as you see fit, and please voice your reactions and thought process aloud. We particularly appreciate critical feedback to improve our interface.**

**I’d like to hear your thoughts about this specific dashboard.**

1. **What did you like about this dashboard?** *Usability*
2. **What could be improved about this dashboard?** *Usability*
3. **How did you feel about the information provided on the dashboard? Were there any items that felt irrelevant, or items you wish you saw?**

**Thank you for your feedback! Please hit the left button until you return to the first screen you saw. I will now read this vignette:**

**Jana D. is a 53 year old female who presents to your clinic for a well visit. She has a history of stroke, myocardial infarction, and three-drug hypertension.**

**Please click ‘Second Dashboard’ on the Home Screen. Again, click the links as you see fit, and please voice your reactions and thought process aloud. We particularly appreciate critical feedback to improve our interface.**

**I’d like to hear your thoughts about this specific dashboard.**

1. **What did you like about this dashboard?** *Usability*
2. **What could be improved about this dashboard?** *Usability*
3. **How did you feel about the information provided on the dashboard? Were there any items that felt irrelevant, or items you wish you saw?**

**I will now ask you about your impressions of the entire tool as a whole, independent of how the information provided was arranged.**

1. **How do you think this PAD screening tool would influence your recommendations to patients?** *Patient needs & resources*
2. **What benefits would you anticipate to using this screening tool? What challenges?** *Acceptability*

**Thank you very much for your time!**
