# Supplementary material for: Physician- and Patient-Elicited Barriers and Facilitators to Implementation of a Machine Learning–Based Screening Tool for Peripheral Arterial Disease: Preimplementation Study With Physician and Patient Stakeholders
Source: JMIR Cardio. 2023 Nov 6;7:e44732. doi: 10.2196/44732 (PMC10660241; doi:10.2196/44732)
Supplement: Multimedia Appendix 2 [file cardio_v7i1e44732_app2.docx]

**PAD ML Interview Guide: Patients**

**RESEARCH QUESTIONS** *(for internal purposes only)*

What are patient perceptions regarding barriers and facilitators to machine-learning based PAD screening implementation, particularly in regards to usability, acceptability, and compatibility with patient needs?

***3 people in a group minimum before identification, otherwise collapse.**

***Let people know who is going to know they participate or not**

**INTRODUCTION**

Hi, my name is (insert name here) and I am calling from Stanford University. I am working with Stanford Clinics to administer a 5-minute survey regarding new technologies in healthcare. This survey is completely optional and will not influence your current care. Would you be interested in participating?

Before we start, everything you say today **will be kept confidential. I am the only person who will be looking directly at transcripts, and will not tell anyone whether you participated or not**.

I would also like to audio record the interview to help me focus on you instead of taking notes. Are you comfortable with me **recording** our confidential discussion?

- Ok, I am **turning on the recorder** now.
- Do you give your **consent** to be recorded to this evaluation?
- Can you **please say your name, date**, and that you **accept being recorded**?
- To be **cognizant of your time**, how long are you able to chat with me today? Do you have a hard stop we need to pay attention to?

1. **We are interested in your thoughts about a condition called peripheral arterial disease, where there is low circulation to the legs. What do you think about peripheral arterial disease?**
2. **We are interested in your thoughts about artificial intelligence, also known as machine learning in healthcare. What do you think about AI or ML in healthcare?**
3. **We are piloting the use of a tool based on artificial intelligence to estimate your risk of having poor circulation to the legs. How do you feel about a physician using this with you?**
4. **Would you feel comfortable making decisions about your health based on this tool?**
5. **Is this something you would talk to your friends about?**

**Thank you very much for your time!**
