# Supplementary material for: Physician- and Patient-Elicited Barriers and Facilitators to Implementation of a Machine Learning–Based Screening Tool for Peripheral Arterial Disease: Preimplementation Study With Physician and Patient Stakeholders
Source: JMIR Cardio. 2023 Nov 6;7:e44732. doi: 10.2196/44732 (PMC10660241; doi:10.2196/44732)
Supplement: Multimedia Appendix 3 [file cardio_v7i1e44732_app3.pdf]

Supplementary Document 3: Template Summary

Participant Code: <redacted>

Theme	Subtheme	Summary	Supporting Quotation
Usability	Visuals	Preference for graphic depictions	I really like the visuals with the pie graph. I think that's nice
	Navigation	Preference for fewer clicks	When you don't know how many more screens there are, it's not as satisfying
			I think it would be more useful to me to see all of the things that were potential guideline options listed, and then I could click on them
	Content	Preference for less information regarding tissue loss, diabetes	For those guidelines, I'm not sure… didn't really seem that there was any kind of aha moment there for me
			Kind of getting us away from the screening of the asymptomatic patient
	Format	Preference for less text	Who's going to be reading this? If it's supposed to be a busy clinician in the office, like spending a minute looking at this? This is I think, too much text?
Facilitators	Physician Knowledge	Self-efficacy in educating others about peripheral arterial disease	One of the reasons I was interested in participating in this study is that I did do a pre clinic teaching module for the faculty and residents several years ago about peripheral arterial disease
		Self-efficacy in performing physical exam related to peripheral arterial disease	I'm probably unusual in that I have an ultrasound device in my office. So I can do screening when I have time, and I've done it a handful of times, but sometimes time precludes.
		Interest in quality improvement and resource management	As a clinician I'm always thinking about choosing wisely, and using resources where we can best get diagnostic benefit.
Barriers	Physician Knowledge	Low perceived usefulness due to high perceived knowledge regarding peripheral arterial disease	[The tool] probably wouldn't affect me significantly. But it may be that I… have a little more knowledge because I wrote a module on this… I probably have a little bit more working knowledge about peripheral arterial disease.
		Low relative priority of PAD compared to coronary artery disease	It's not like coronary disease, where if you miss it, and somebody, somebody is going to have an acute event, and you know, death could ensue, right? Whereas if you have peripheral vascular disease that you haven't picked up and they're not symptomatic with it. Is it really going to make a big difference? If you find it earlier? You know, is there is that going to impact

			behavioral change? Will it help them maybe, with their smoking cessation efforts, perhaps.
	Perceptions of machine learning	Negative prior experience with machine learning in healthcare	One of the things that's recently started at Stanford is the AI device for screening retinopathy. The problem is that 70% or more of the patients I send in get an insufficient rating and have to get a full eye exam anyway. So it’s really not performing well.
